# Comparative appraisal of nutrient recovery, bio-crude, and bio-hydrogen production using *Coelestrella* sp*.* in a closed-loop biorefinery

**DOI:** 10.3389/fbioe.2022.964070

**Published:** 2022-09-23

**Authors:** Harishankar Kopperi, S. Venkata Mohan

**Affiliations:** ^1^ Bioengineering and Environmental Sciences (BEES) Lab, Department of Energy and Environmental Engineering, CSIR-Indian Institute of Chemical Technology (CSIR-IICT), Hyderabad, India; ^2^ Academy of Scientific and Innovative Research (AcSIR), Ghaziabad, India

**Keywords:** nutrient recovery, aliphatic/aromatic hydrocarbons, flat-panel photo-bioreactor, circular chemistry, acidogenesis/dark-fermentation, decarbonization, semi-synthesis

## Abstract

A closed loop algal-biorefinery was designed based on a three-stage integration of dairy wastewater (DWW) treatment, hydrothermal liquefaction (HTL) of defatted algal biomass, and acidogenic process in a semi-synthetic framework. Initially, *Coelestrella sp* SVMIICT5 was grown in a 5 L photo-bioreactor and scaled up to a 50 L flat-panel photo-bioreactor using DWW. The microalgal growth showed higher photosynthetic efficiency, resulting in a biomass growth of 3.2 g/L of DCW with 87% treatment efficiency. The biomolecular composition showed 26% lipids with a good fatty acid profile (C_12_-C_21_) as well as carbohydrate (24.9%) and protein (31.8%) content. In the second stage, the de-oiled algal biomass was valorized via HTL at various temperatures (150°C, 200°, and 250°C) and reaction atmospheres (N_2_ and H_2_). Among these, the 250°C (H_2_) condition showed a 52% bio-crude fraction and an HHV of ∼29.47 MJ/kg (bio-oil) with a saturated hydrocarbon content of 64.3% that could be further upgraded to jet fuels. The energy recovery (73.01%) and elemental enrichment (carbon; 65.67%) were relatively greater in H_2_ compared to N_2_ conditions. Finally, dark fermentation of the complex-structured HTL-AF stream resulted in a total bio-H_2_ production of 231 ml/g of TOC with a 63% treatment efficiency. Life cycle analysis (LCA) was also performed for the mid-point and damage categories to assess the sustainability of the integrated process. Thus, the results of this study demonstrated comprehensive wastewater treatment and valorization of de-oiled algal biomass for chemical/fuel intermediates in the biorefinery context by low-carbon processes.

## Graphical Abstract

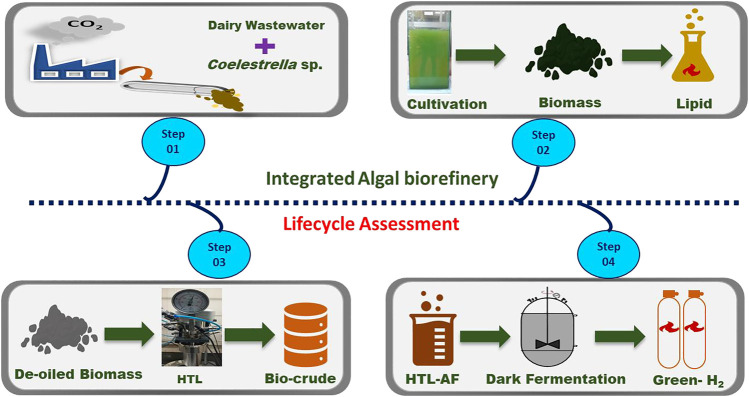

## Highlights

• *Coelestrella* sp. SVMIICT5 was used for dairy wastewater treatment and nutrient recovery
• Photosynthetic transition and biomolecular yields during microalgae cultivation were studied
• Bio-crude, aqueous fraction, and energy profiles during HTL were studied
• A higher bio-crude fraction (52%) was observed in H-HTL at 250°C
• The bioprocess (HTL-AF) resulted in 231 ml/g of bio-H_2_ with 63% TOC removal
• Microalgae with a semi-synthesis approach allowed closed-loop biorefinery

## 1 Introduction

Biomass-based biorefineries have recently been considered as a potential strategy to mitigate environmental pollution and climate change by ensuring sustainable waste management (Venkata Mohan et al., 2020; Hao et al., 2021; Wahab et al., 2022). Bio-based energy production is one sustainable alternative (Amulya et al., 2020; Kokkinos et al., 2021; Kopperi et al., 2021), accounting for 9–10% of the global energy supply (Bagchi et al., 2021). Using nature-inspired processes to design an efficient biorefinery system to produce environmentally-friendly biofuels and chemicals can help to build sustainable bio-refineries and carbon-neutral bioeconomies (Katakojwala and Venkata Mohan, 2021). Microalgae is emerging as a third-generation feedstock for the production of biofuels with high energy density (Galadima, and Muraza, 2018; Mutanda et al., 2020; Bagchi et al., 2021) with in-built bio-sequester (CO_2_) capability and the accumulation of significant lipids and other value-added products (Chew et al., 2017; Kokkinos et al., 2021; Kuravi and Mohan, 2021). Algal biorefinery is a potential platform that has been studied extensively for the production of a variety of products in a sequential and integrated pathway (Venkata Mohan et al., 2020). Following wastewater treatment, bio-oil extraction from microalgae, targeting biogas production by utilizing biomass, defatted biomass for improving soil fertility, etc. has been explored (Prajapati et al., 2014; Venkata Mohan et al., 2020; Arora et al., 2021). Phycoremediation uses nutrients as a growth medium to provide biomass production (Venkata Mohan et al., 2020). Nutrient recovery using algae followed by integration with an aerobic digester for the production of biofuels in closed-loop approaches has been reported (Prajapati et al., 2014; Al-Jabri et al., 2020). The bioremediation of various wastewaters (marine, brackish water, etc.), as well as CO_2_ fixation from CO_2_-rich flue gas emissions from stationary using algae, have also been evaluated (Hemalatha et al., 2019; Molazadeh et al., 2019; Watson et al., 2020).

Thermo-chemical processes such as gasification, pyrolysis, and hydrothermal liquefaction (HTL) are being considered as efficient routes to rapidly convert biomass to fuels or chemicals (Wang et al., 2018; Kokkinos et al., 2021). HTL has emerged as an economically feasible and environmentally friendly method for biomass conversion. HTL uses water as a catalyst to convert algae biomass to bio-crude and other by-products in an inert or reducing system (oxygen-free) at elevated temperatures and high pressures of 5–28 MPa (Katakojwala et al., 2020; Hao et al., 2021; Kopperi et al., 2022). Process conditions such as solid-to-liquid ratio, feedstock composition, temperature, solvent, pressure, and catalyst impact the efficiency and specificity of the reaction products (Carpio et al., 2021). The practical application of HTL for converting organic feedstock into biofuels and further upgradation resulting in aviation/jet fuels with zero waste is of increasing interest in the global community (Hao et al., 2021; Kopperi et al., 2022). However, HTL results in an aqueous organic fraction of 20–50% (HTL-AF) from feedstock and its recovery from AF is an important step for nutrient and energy recovery (Si et al., 2019; Watson et al., 2020). The HTL-AF contains complex molecules including furans, phenols, volatile fatty acids (VFAs), N-heterocyclic compounds, etc. Moreover, the integration of dark fermentation (DF) is a potential pathway for bio-energy production due to its higher tolerance for HTL-AF. It is also a potentially cost-effective biological process with reduced environmental impacts (Watson et al., 2020; Chen et al., 2021: Kopperi et al., 2022).

The chemical industry is currently reorienting itself towards semi-synthesis employing biobased materials or combining chemicals with renewable feedstocks (Venkata Mohan and Katakojwala, 2021). In this context, the integrated biorefinery process is a semi-synthetic route for the conversion of biological feedstock to valuable energy/fuel and chemical components. Additionally, the utilization of the byproducts could ensure the overall sustainability of the process, ensuring the circular chemistry paradigm shifting from conventional linear flow practices to low-carbon closed-loop approaches (Katakojwala and Venkata Mohan, 2021; Kopperi et al., 2022). Therefore, the present study mixotrophically cultivated *Coelestrella* sp. using dairy wastewater with an integrated strategy in the “waste to wealth” framework. Initially, this study explored wastewater treatment, nutrient recovery, and biomass growth with photosynthetic response. Bio-molecules (carbohydrates, proteins, and lipids) accumulation was also studied in detail. Additionally, HTL was applied to produce bio-crude from the defatted biomass under different temperatures and atmospheric conditions. The biomass conversion ratios, bio-crude yields, and energy recovery ratios were explored. Furthermore, HTL-AF carbon and nutrient recovery were studied by DF to determine the bio-H_2_ for all HTL experiments using pretreated mixed microbial consortiums. Furthermore, a life cycle assessment of the integrated algal biorefinery was performed based on the “cradle to gate’’ system boundary including the sub-process steps of algal cultivation in dairy wastewater, biomass de-oiling (lipid extraction), bio-oil recovery, and bio-H_2_ production. Based on the above descriptions, the waste valorization and resource recovery using microalgae were evaluated in a circular biorefinery approach.

## 2 Materials and methods

### 2.1 Microalgae strain

**FIGURE 1:**
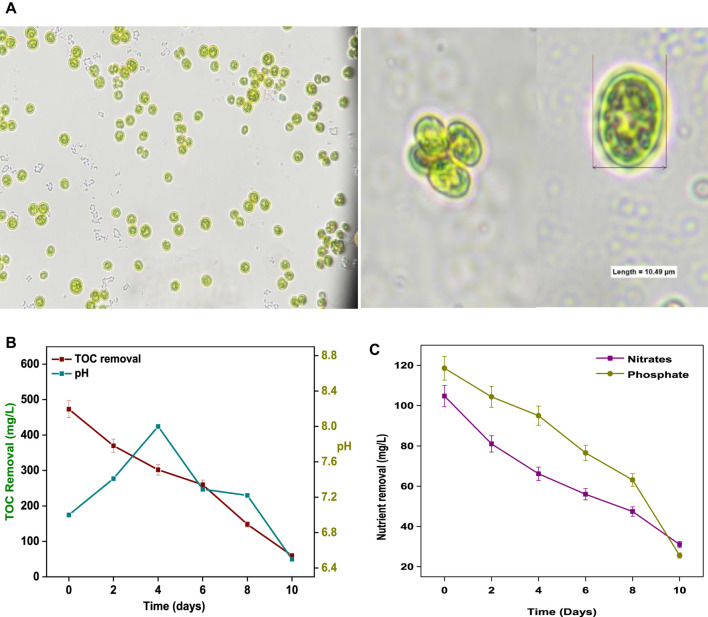
**(A)** Microscopic images and **(B)** size and shape of *Coelestrella* sp. **(B)** TOC removal and pH change and **(C)** nutrient removal with respect to time during SDWW treatment.

Indigenous *Coelastrella* sp. SVMIICT5 microalgae were isolated from water body at CSIR-IICT, Hyderabad (17.4301° N, 78.5416° E) by quadrant streaking. Microscopic observations showed *Coelastrella* sp. cells arranged in tetraploid and hexaploid (four and six cells) structures. Each cell was oval in shape and >10 µm in size (Figure 1A). The isolate was deposited at the National Collection of Industrial Microorganisms (NCIM) in Pune (accession number NCIM-5793 (05-04-2021)).

### 2.2 Flat-panel photo-bioreactor-mixotrophic cultivation

Initially, *Coelastrella* sp was pre-cultured in a 50 ml tube in 30 ml modified sterile 3 N-Basel Bold Medium (BBM) (Kona et al., 2021) at 25 ± 1°C for 7 days. Later growth was continued in 250 ml flasks and optimized. The culture was further scaled in 5 L Erlenmeyer flasks in 3.5 L of 3N-BBM for 7 days with a pH of 7.0. Continuous air bubbling was provided to avoid cell aggregation After 7 days, the cells were harvested for further experiments. Mixotrophic (axenic) outdoor cultivation was performed in 50 L (working volume) flat panel photo-bioreactors (FP-PBR; 45 cm (length) x 15 cm (width) x 90 cm (height); 8 mm (thick) transparent glass) exposed to natural sunlight (ambient temperature, 29 ± 6°C). The reactor was fixed with fine bubbling air spargers at the bottom. Continuous air was supplied using an air pump (Hailea-Aco-208) at a flow rate of 35 L/min to provide CO_2_ and to mix the culture. The composition of the synthetic dairy wastewater (SDWW) was as described elsewhere, with minor modifications (Kiran and Mohan, 2022) (Table 1). FP-PBR fed with 40 L of SDWW was inoculated with 10% C*oelestrella* sp SVMIICT5 (OD, 0.1) and cultured for 10 days. The reactor was monitored by microscope to observe the cell purity and size. During microalgal growth, samples were collected on alternate days and centrifuged (7000 RPM; 5 min) to determine the pH; biomass concentration; carbon, nitrogen, and phosphate removal; and bio-molecular composition.

**TABLE 1:** Chemical composition of the synthetic dairy wastewater (SDWW).

Ingredient	Amount (gL^−1^)
Skim milk powder	0.8
Urea	0.27
CH_3_COONa	0.21
K_3_PO_4_	0.15
(NH_4_)_2_SO_4_	0.06
MgSO_4_·7H_2_O	0.05
NH_4_Cl	0.54
Na_2_HPO_4_⋅2H_2_O	0.9
NaHCO_3_	1.56
KCl	0.6
CaCl_2_⋅H_2_O	0.036
TOC	0.473
NO_3_^−^	0.104
PO_4_^3-^	0.118

### 2.3 Hydrothermal liquefaction-de-oiled biomass

The de-oiled algal biomass (DAB; lipid extracted) was dried and used as a feed for the HTL reaction using deionized (DI) water as the solvent. A 15% (w/v) of DAB was mixed in 200 ml of DI water and transferred to a customized 300 ml high-pressure stirred reactor (KLB Instrument Ltd., India). Initially, the reactor headspace was purged with inert gas (N_2_) to replace the air and further pressurized to 100 bar with N_2_ (N-HTL) or H_2_ (H-HTL) to create inert and reduced atmospheres, respectively. The reactions were performed at various (150, 200, and 250°C) for inert (N-HTL) and reduced (H-HTL) conditions for 60 min with vigorous mixing (500 rpm). After 60 min of reaction, ice-cold water was passed through the cooling coil to stop further reactions. The gases from the headspaces were collected into Tedlar gas bags and analyzed. The solid and liquid fractions were filtered and the solid residue was washed with acetone/chloroform to remove organic soluble mater and dried at 105°C for 12 h. The conversion yields were calculated as the weight losses during the experiments. Liquid-liquid separation methods were used using an organic solvent (chloroform) to separate the aqueous and organic fractions of the bio-crude.

### 2.4 Dark fermentation-bio-H_2_ production

The HTL-AF from all experiments was used as feedstock for bio-H_2_ production using the pre-treated inoculums (Santhosh et al., 2021) taken from the semi-pilot scale bio-hydrogen reactor at CSIR-IICT. The experiments were performed in 500 ml bioreactors with 400 ml working volume and 100 ml of headspace with a retention time of 72 h at 35 ± 2°C. The carbon content of HTL-AF was adjusted to 4 g/L of TOC and a pH of 6.5 Gas production was monitored in a continuously stirred bioreactor (Bioprocess Control- AMPTS II, Sweden). The gas outlets from the bioreactors were connected to a flow meter through fixed gas lines that continuously measured biogas in an online system. The gas production and substrate conversions were evaluated by sampling liquid and gas at 12-h intervals and analyzed further.

### 2.5 Analysis

#### 2.5.1 Microalgae growth

Algal growth was quantified by UV-VIS spectrophotometer (JASCO V-750) at a wavelength of 720 nm to measure biomass growth. The dry cell weight (DCW) was obtained by passing culture through filter paper and drying it in an oven at 60°C until the biomass was invariant. The specific algal growth rate (mg d^−1^) was calculated using Eq. 1, where ln X was the n-log of the final DCW and X_0_ was the natural logarithm for the initial DCW at a given interval “t” (Kiran and Mohan, 2021).

$$\text{Specific}\;\text{growth}\;\text{rate}\left(\mu \right)=\text{In}\;\text{X}-\text{In}{\text{X}}_{\text{0}}/\text{t}$$ (1)

#### 2.5.2 Bio-molecule estimation

The samples were measured for pH variation (Adwa, AD-8000). The nitrate and phosphates (mg L^−1^) removal was measured by standard protocols (American Public Health Association and American Water Works Association, 1998). The chlorophyll a, b and carotenoid contents of the algal biomass were estimated by cell disruption (40 kHz; 5 min) with 90% acetone and the supernatant was separated (7000 xg). The chlorophyll (a, *b*) and carotenoid concentrations in the supernatant were measured based on their ODs at 661.6, 644.8, and 470 nm, respectively, and further calculated using Eqs 2–4 (Jeffrey and Humphrey, 1975).

$$\text{Chl}\;\text{a}\left(\mu \text{g}/\text{ml}\right)=11{\text{.}24\text{×A}}_{661\text{.}6}-2{\text{.}04\text{×A}}_{644\text{.}8}$$ (2)

$$\text{Chl}\;b\left(\mu \text{g}/\text{ml}\right)=20{\text{.}15\text{×A}}_{664\text{.}8}\mathrm{-4}{\text{.}19\text{×A}}_{661\text{.}6}$$ (3)

$$\text{Carotenoids}\left(\mu \text{g}/\text{ml}\right)=\left(1000\times {\text{A}}_{470}-1.90\times \mathrm{Chl}\;\mathrm{a-63}\mathrm{.14}\times \mathrm{Chl}\;b\right)/214$$ (4)

The total carbohydrate content was measured using the hydrolysis (phenol-sulphuric acid) method, (Yirgu et al., 2020) while the protein content was estimated using a bicinchoninic acid (BCA) protein assay kit (Takara-T9300A) with bovine serum albumin (BSA) as the standard. The total organic carbon (TOC) content of the SDWW was analyzed on a TOC analyzer (TOC-L CPH, Shimadzu; 4 μg/L to 30,000 mg/L detection limit). The fatty acid methyl ester (FAME) profiles were analyzed using 100 mg of dried biomass (transesterified) to which acidified (2%) methanol was added in a parallel synthesis reactor (Radleys, UK) and kept at 70°C for 5 h (close-refluxing). The mixture was fractionalized using a 2:1 ratio of ethyl acetate and water. The lipid product was dissolved in anhydrous chloroform for gas chromatography (GC; (Agilent- 7890B) analysis (Kuravi and Mohan, 2021).

#### 2.5.3 Photosynthetic fluorometry measurements

The Fv/Fm (PS I) parameters were measured by fluorescence using a PAM fluorometer (Aqua pen, AP-C 100) on whole algal cells adapted to the dark for 10 min (Kona et al., 2021). The photosynthetic regulatory reactions of *Coelastrella* sp were studied based on the PSII (P680-specific *Chl a* fluorescence) and PSI (P700-specific light absorption) signals using a DUAL-pulse amplitude modulator (DUAL-PAM; Walz, Germany). For these measurements, 1 ml of thick algae culture was dropped in a cuvette (quartz-10 mm) under continuous stirring with a micro stirrer and adapted in the dark for 15 min to open the reaction centers (RCs) of the photosystems (PSII and PSI) before measuring (White et al., 2011). The rapid light curve (RLC) triggers were measured every 10 s, escalating actinic irradiance from 10–832 μmol photons m^−2^s^−1^. The RLCs provided a snapshot of electron transport chain (ETR) saturation and the overall photosynthetic performance of the *Coelastrella* sp. The dual PAM was operated with v-1.9 software to record the data and generate RLCs (White et al., 2011; Jokel et al., 2018; Kiran and Mohan, 2021).

#### 2.5.4 HTL and bioprocess analysis

The off-gas composition was assessed by GC (Agilent- 7890B). The elemental (C, N, S, H, and O) compositions of solid and liquid (biochar, bio-oil) samples were analyzed (ElementarVario Microcube-63505). The bio-oil profiles were identified by GC-MS. The chromatogram peaks were analyzed using the NIST-Database (Katakojwala et al., 2020). The aqueous fraction composition was analyzed by high-resolution quadrupole time-of-flight mass spectrometry (HRMS; Waters AcquityXevo G2-XS) (Katakojwala et al., 2020). The DF samples were analyzed three times using TOC. The average results were presented. The VFA composition was estimated by high-performance liquid chromatography (HPLC; Shimadzu LC20A) (Kopperi et al., 2022).

##### 2.5.4.1 Yield calculations

The DABs for various constituent conversions such as bio-crude yields (%), HTL conversion (%), and higher heat value (HHV, MJ.kg^−1^) were calculated using Eqs 5–8 (Villaver et al., 2018; Wang et al., 2018). Elements (E) (C, H, N, S, and O) and HHV of DAB and bio-crude were calculated based on Eq. 6. The bio-crude yields and HTL conversions were calculated according to Eqs 5 and 7. From the equations, m_B_, m_A_, m_M_, m_SR_, and m_C_, are the mass of the bio-crude, ash of algae, microalgae, solid residue, and catalyst, respectively. The energy recoveries (ER) were the HHV ratios of the bio-crude to DAB-HTL and are calculated using Eq. 8, where HHV_M_ and HHV_B_ are the HHVs of microalgae and bio-crude, respectively.

$$\text{BiocrudeYield}\left(\text{\%}\right)=\frac{{\text{m}}_{\text{B}}}{{\text{m}}_{\text{M}}}\text{×}100\text{\%}$$ (5)

$$\text{HHV}\left({\text{MJKg}}^{-1}\right)=\text{0}{\text{.}3404\text{C}}_{\text{B}}+1{\text{.}2432\text{H}}_{\text{B}}+\text{0}{\text{.}0628\text{N}}_{\text{B}}+\text{0}{\text{.}1909\text{S}}_{\text{B}}-\text{0}{\text{.}0984\text{O}}_{\text{B}}$$ (6)

$$\text{HTLConversion}\left(\text{\%}\right)=\left\{1-\frac{{\text{m}}_{\text{SR}}{-\text{m}}_{\text{A}}{-\text{m}}_{\text{C}}}{{\text{m}}_{\text{C}}}\right\}\text{×}100\text{\%}$$ (7)

$$\text{EnergyRecovery}\left(\text{\%}\right)=\frac{{\text{HHV}}_{\text{B}}\text{×BiocrudeYield}}{{\text{HHV}}_{\text{M}}}$$ (8)

### 2.6 Life cycle analysis

The environmental sustainability of integrated algal biorefinery with a defined system boundary was analyzed using LCA software (SimaPro v.9.1.1) as per ISO 14040:2006 guidelines. The inventory for energy and chemical inputs to the biorefinery system are detailed in Figure 6A. A cradle-to-gate system boundary approach was applied, with a functional unit of a 100 L biorefinery system, including dairy wastewater treatment, biomass processing (lipid extraction/de-oiling), HTL, and acidogenesis. (Supplementary Table S2). All the primary inputs, as well as secondary data, were provided according to the LCA framework (Katakojwala and Venkata Mohan, 2021). The best experimental conditions (H-HTL-250°C) were considered in the LCA study. The sustainability of the integrated biorefinery process was studied with respect to fifteen mid-point and four end-point (damage) categories using the Impact 2002 + lifecycle impact assessment (LCIA) method. The damage impact categories included health, ecosystem, quality, climate change, and resource depletion (Sarkar et al., 2021).

## 3 Results and discussion

### 3.1 Cultivation and metabolites

#### 3.1.1 Wastewater treatment

During cultivation, changes in nutrients (N and P), pH, and carbon were monitored (Figure 1). The pH increased from 7.1 (day 1) to 8.02 (day 4) and then gradually decreased to 6.59 (day 10) (Figure 1B). The pH influences microalgae cultivation by affecting nutrient uptake. The nitrate and phosphate concentrations decreased from 104 mg/L and 118 mg/L, respectively, to 31 mg/L and 25 mg/L with removal efficiencies of 70.39 and 80.1% by the end of day 10 (Figure 1C). The nutrient (N and P) uptake by microalgae occurred through interconnected biochemical pathways for storage/assimilation into nucleic acids and proteins for cell growth (Taziki et al., 2015). Nitrates were assimilated into amino acids and processed for protein formation (Taziki et al., 2015). Phosphorus (as orthophosphate) entered the cell membranes and was assimilated into nucleotides for ribosomal RNA synthesis (Mao et al., 2021). The levels of organic carbon (TOC) decreased from 473 mg/L to 60 mg/L by the 10th day of cultivation, with 87% treatment efficiency (Figure 1B).

#### 3.1.2 Biomass and pigments

**FIGURE 2:**
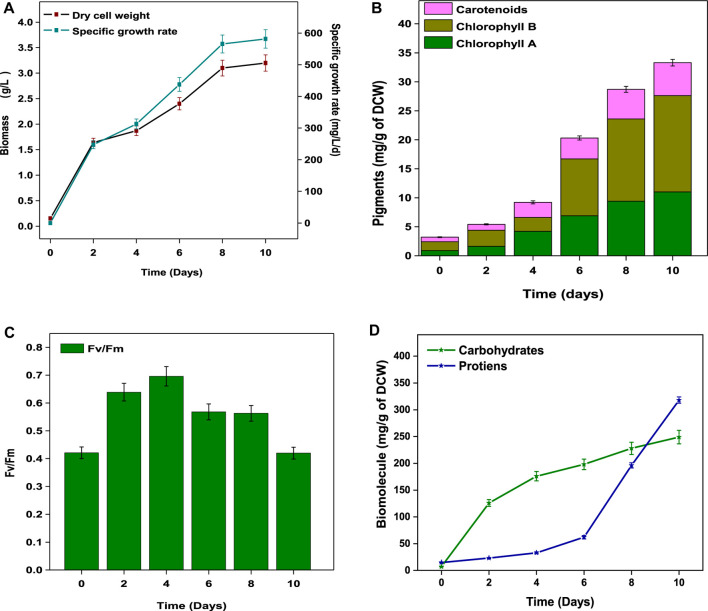
**(A)** Maximal photosynthetic yield (Fv/Fm). **(B)** Biomass and specific growth rate. **(C)** Chlorophyll a, b, and carotenoids and **(D)** total carbohydrate and protein contents.

During the experimental period, the growth characteristics of *Coelestrella* sp were monitored every two days. The SDWW was inoculated with 0.15 g/L (DCW) (day 0) of microalgae culture. The concentration increased to 3.2 g/L (DCW) by the end of the growth period (day 10). The microalgae growth with organic carbon under mixotrophic conditions increased the growth rate by improving biomass yield and lipid accumulation, simultaneously consuming CO_2_ and producing oxygen through photosynthesis (Lv et al., 2019). SDWW treatment (carbon removal) was well correlated with the biomass growth rate. A specific growth rate of 581.57 mg/L/d was observed on the 10th day of cultivation (Figure 2A). Similar growth and carbon removals fractions were previously performed with *Tetradesmus* sp. and *Scenedesmus* sp. cultures using different wastewater (Alvarez-Diaz et al., 2015; Kiran and Mohan, 2022). Elemental analysis of the harvested biomass showed 48.1% C, 6.3% H, 9.5% N, 0.5% S, and 35.6% O. No growth inhibition was observed in the SDWW during the cultivation period. The tolerance of diverse wastewaters for the cultivation of microalgae has been reported (Al-Jabri et al., 2020). Kothari and co-workers (Kothari et al., 2013) reported no toxic or inhibitory effects on microalgae growth when dairy wastewater (maximum 6 g/L) was used as cultivation medium. However, the algal growth rates were lower in industrial wastewaters due to the presence of toxic metal ions such as Cd, Cr, etc. and organic toxins (hydrocarbons, surfactants, biocides, etc.) (Molazadeh et al., 2019).

The pigment fractions (chlorophyll (*Chl*) a and b and carotenoids) as a function of cultivation time are shown in Figure 2B. Gradual increases in *chl* a and *chl* b concentrations were observed from day 4 to 10 and reached maximum concentrations of 11 mg/g (*chl* 1) and 16.6 mg/g (*chl* 2) by the end of the cycle (day 10). The concentrations of secondary metabolite (carotenoids) increased significantly from 0.8 mg/g (day 0) to a maximum value of 5.7 mg/g on day 10. The chlorophyll content is directly proportional to biomass growth (Masojídek et al., 2010) with the conversion of 5-aminolevulinic acid to porphobilinogen (Huang et al., 2019). Intracellular free nitrogen (nitrates, amino acids) directs the controlling metabolism toward carotenoid synthesis (Huang et al., 2019). The changes in the photosynthetic activity of *Coelestrella* sp. were also analyzed. The Fv/Fm ratios indicate the PSII (photosystem) photochemical efficiency in the dark-adapted state with fully open reaction centers of the PSII system (Kona et al., 2021). Initially, the Fv/Fm value was 0.42 (day 0) and later reached a maximum value (0.69) on day 4, indicating a maximum photosynthetic activity. The ratio then gradually decreased to 0.42 by the end of cultivation (Figure 2C). The lower Fv/Fm ratios might result in carotenoid synthesis (Masojídek et al., 2010).

#### 3.1.3 Bio-molecules

Analysis of the biomolecule composition of *Coelestrella* sp. showed 24.9% carbohydrates, 31.8% proteins, and 26% total lipids (10% neutral lipids) (Table 2; Figure 2D). A maximum concentration of 249 mg/g of carbohydrate was observed on day 10. The protein fraction resulted in 318 mg/g of biomass at the end of the cycle. Algae synthesize carbohydrates by photosynthesis-mediated carbon absorption of cells by an inducible (hexose/H^+^) symport mechanism (Alvarez-Díaz et al., 2015). Carbon-rich SDWW results in an easy uptake of carbon compared to other sources for carbohydrate synthesis. The nutrients (N and P) and carbon sources direct metabolism toward protein synthesis and cell growth acceleration. In the presence of adequate carbon source and light energy, proteins are further utilized and converted to carbohydrates or lipids (Hemalatha et al., 2019). The photosynthetic carbon internalization mechanism shifts from the production of molecules such as proteins and carbohydrates to the storage of lipids (Alvarez-Díaz et al., 2015). A C/N imbalance in the cell caused due to nitrogen deprivation affects metabolism, promoting the storage of lipids/triglycerides (Kuravi and Mohan, 2021).

**TABLE 2:** Composition analysis of *Coelestrella* sp. SVMIICT5 after cultivation.

Component	Unit fraction
Biomass yields (DCW)	3.2 ± 0.16 (g/L)
Specific growth rate	0.43 ± 0.02 (g/L/d)
Carbohydrate	24.9 ± 1.24%
Protein	31.8 ± 1.5%
Total Lipid	26 ± 1.3%
Neutral Lipid	10 ± 0.5%
Fatty acids Composition	Fatty acid (%)
Undecanoic acid (C11:0)	2.2 ± 0.1
Lauric acid (C12:0)	4.6 ± 0.2
Tridecanoic acid (C13:0)	5.9 ± 0.3
Myristic acid (C14:0)	7.3 ± 0.35
Pentadecanoic acid (C15:0)	6.2 ± 0.3
Heptadecanoic acid (C17:0)	11.5 ± 0.5
Arachidic acid (C20:0)	4.3 ± 0.21
Myristoleicc acid (C14:0)	8.6 ± 0.4
Pentadecanoic acid (C15:1)	14.1 ± 0.7
Heptadecanoic acid (C17:1)	16.9 ± 0.8
Oleic acid (C18:1)	8.6 ± 0.43
Linolenic acid (C18:2 *ω*-6)	3.5 ± 0.17
Eicosapentanoic acid (C20:5ω-3)	6.3 ± 0.3
SFA	**50.6** ± 2.5
MUFA	**39.6** ± 1.9
PUFA	**9.8** ± 0.49

When assessing the commercial viability of microalgae, lipid productivity is one key product. The *Coelestrella* sp. dried biomass resulted in 26% of total lipids per gram of DCW, with 10% neutral lipids. The GC-FAME analysis showed a wide range of saturated (SFA) and unsaturated (USFA) fatty acids. The fatty acid profiles of *Coelestrella* sp. SVMIICT5 showed a relatively higher fraction of SFA (50.6%) followed by USFA (49.4%) (Table 1). In SFA, heptadecanoic acid (C17:0) was a major fraction (11.5%), followed by myristic acid (C14:0, 7.3%) and pentadecanoic acid (C15:0, 6.2%). The USFA contained monounsaturated fatty acids (MUFAs) such as pentadecanoic acid (C15:1, 14.1%), heptadecanoic acid (C17:1, 16.9%), and oleic acid (C18:1, 8.6%). The polyunsaturated fatty acids (PUFAs) included 3.5% linolenic acid (C18:2 *ω*-6) and 6.3% eicosapentaenoic acid (C20:5ω-3). By the end of the treatment, the nutrient deficiency and high irradiance favored higher MUFA assimilation (Kuravi and Mohan, 2021). The above-mentioned fatty acids have medicinal and biotechnological applications in fuels, nutrition, fodder, pharmaceuticals, and skin care products (Kiran and Mohan, 2022). Linolenic acid in PUFA is most useful in the synthesis of eicosapentaenoic acid (EPA) and docosahexaenoic acid (DHA), which reduce the risk of immunological, neurological, and degenerative diseases (arthritis, heart, and skin) (Kuravi and Mohan, 2021). SFA and USFA are commonly used as emulsifiers in skin care, antioxidant, antibacterial agent, and lubricant preparations (Hemalatha et al., 2019; Kuravi and Mohan, 2021). The mixotrophic growth of *Coelestrella* sp. SVMIICT5 showed significant biomass production and lipid productivity, with fatty acid composition associated with nutraceutical functions and lubricant preparations.

### 3.2 Photosynthetic transients

**FIGURE 3:**
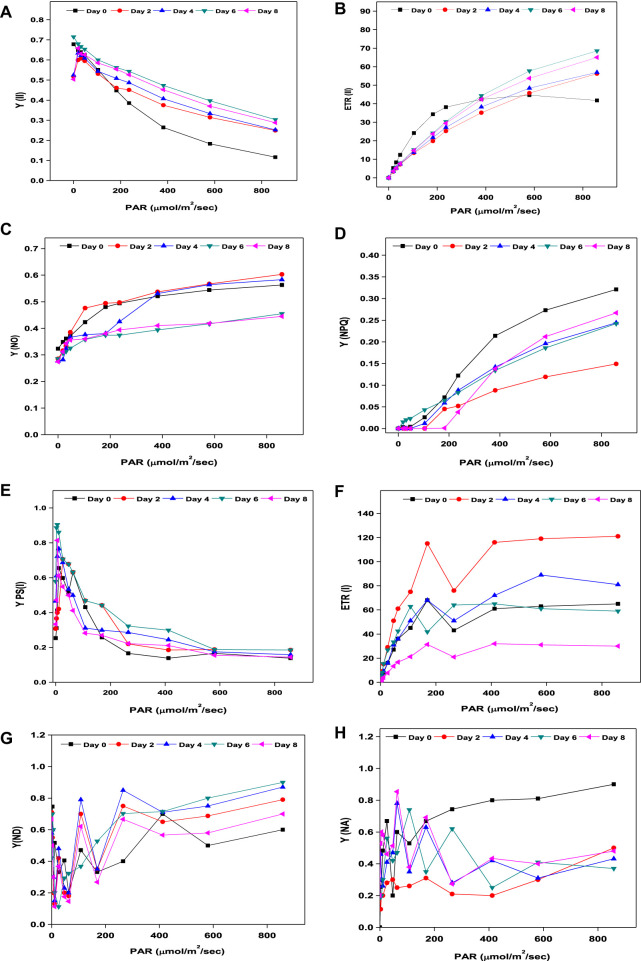
**(A–D)** Chlorophyll a fluorescence. **(E–H)** P700 transients of *Coelestrella* sp.

On every other day from day 0 to 8 during the growth period, photosynthetic parameters (PSII and PSI) of *Coelestrella* sp. SVMIICT5 were measured immediately after 10 min of dark adaptation (Figure 3). Dark adaptation relaxes the thermal dissipation mechanism and oxidizes photosynthetic reaction centers, resulting in maximum photochemical efficiency (Fm). Upon activation of actinic light (AL) on chlorophyll pigments, chlorophyll a fluorescence and P700 transients aid in the measurement of photosynthetic performance (Kiran and Mohan, 2022). PS II is explained by Fo, Fm, F′, and Fm’ (fluorescence variables), whereas the energetic state (PSI) is defined according to the Po, Pm, and Pm’ fluorescence variables (Kona et al., 2021). While PSII and PSI have different light absorption maxima, activating their light-harvesting centers enhances their respective photosystems. YPSII determines both electron excitation to drive PSII and PSI re-opening after photochemical activity (Bonnanfant et al., 2019). By induced saturation pulse, the maximal PSII quantum yield in the present study increased from 0.518 (day 0) to 0.715 (day 6), indicating improved photosynthetic performance, before gradually decreasing to 0.504 (day 8) (Figure 3A). The splitting of water molecules in PSII is facilitated by the light energy captured by reaction centers (RCs), with the released protons driving PSI to reduce ATP and NADPH (Bonnanfant et al., 2019). The increased YPSII ratios indicated efficient electron donors at the initial and final electron acceptors, implying faster cell growth until day 6 in SDWW (Kiran and Mohan, 2021). The ETRII is a measure of electron transport in the photosynthetic cycle, which is also aligned with oxygen liberation and CO_2_ fixation. The electron transfer rate of *Coelestrella* sp. cells increased steadily from day 0 (41.7) to day 6 (68.5) and decreased slightly to 65 at end of day 8 of the cycle upon induction with actinic light (Figure 3B). This decrease might be caused by excessive exposure to light or nutritional depletion during the growth process (Bonnanfant et al., 2019). A higher Fv/Fm ratio implies the efficiency of water splitting and carbon fixation at PSII, allowing for higher photosynthetic electron transport (Wimalasekera, 2019).

Non-photochemical quenching drives microalgae to lose surplus light energy as heat and fluorescence from light-harvesting complexes, which is inversely proportional to quantum yield [Y(NPQ) - regulated energy dissipation and Y(NO) - non-regulated energy dissipation] (Tikkanen et al., 2015). Y(NO) (inversely proportional to YPSII) decreased from 0.563 (day 0) to 0.445 (day 8) (Figure 3C). As the cell growth increases, Y(NO) remains steady and defective in inducing thermal dissipation (Wimalasekera, 2019). The change in Fm and Fm’ indicates photochemical quenching (NPQ). An increase from 0.149 (day 2) to 0.267 (day 8) was observed (Figure 3D). The PSI photosynthetic metrics included Y(I), ETR(I), Y(ND), and Y(NO). The photochemical quantum yield of PSI increased from 0.253 (day 0) to 0.578 (day 6) and decreased slightly from day 8 (Figure 3E). The electron transport rate ETR(I) of PSI showed a maximum rise from 2.2 to 61 on day 6 and further decreased to 31 by the end of the treatment period (Figure 3F). Y(ND) was inversely proportional to Y(I). By the end of the growth period, there was a study decrease in Y(ND) from day 0 (1.0) to day 8 (0.66) (Figure 3G). The redox potential of the intersystem electron transport chain controls the PSI acceptor side limitation, Y (NA). In the present study, NA decreased with increases in PAR, from 0.81 (day 0) to 0.4 (day 8) (Figure 3H). As a result, sufficient electron and proton transport, which is required for ATP and NADPH generation under limited conditions, restricted the photosynthetic assimilation, which further affected biomass and biomolecules (Bonnanfant et al., 2019).

### 3.3 HTL conversion and yields

#### 3.3.1 Conversion profiles

**FIGURE 4:**
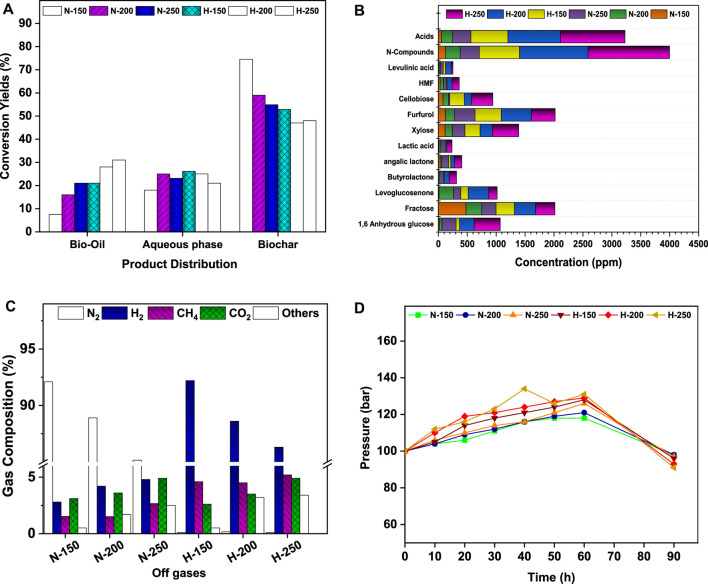
**(A)** Product distribution profile. **(B)** Aqueous fraction product profile. **(C)** Off-gas composition. **(D)** Pressure variation with respect to temperature.

The liquefaction of de-oiled biomass resulted in bio-crude i.e., aqueous and bio-oil fractions, along with the co-products. The yield and composition of the respective products were measured for the quantitative carbon flux in the HTL process with reference to temperature and micro-atmosphere. Figure 4A presents the substrate-to-product selectivity (%) on a weight basis towards bio-oil, aqueous fraction, and char at 150°C, 200°C, and 250°C for the N-HTL and H-HTL reactions. With increasing temperature, the yields of bio-crude fraction increased from 12.75 to 44.1% in N-HTL and from 47 to 52% in H-HTL. Comparatively higher bio-oil yields were obtained in H-HTL (150°C: 21%; 200°C^:^ 28%; 250°C^:^ 31%) compared to N-HTL conditions, while the aqueous fractions were higher in N-HTL. The inverse trends were observed for aqueous to bio-oil fractions with increasing temperature. Increasing temperature favors cleavage and recombination reactions between polar organic (water soluble) compounds, resulting in the elimination of polar functional groups, which leads to the formation of bio-crude (water-insoluble) molecules (Xu and Savage, 2017. The char fractions of N-HTL conditions decreased with increasing temperature (150°C: 74.5%, 200°C: 59%, 250°C: 54.9%. The H-HTL conditions showed relatively lower char fractions (150°C: 52.8%, 200°C: 48%; 250°C: 47%). Thus, the char formed in HTL reactions acted as co-products and can be used for trace metal removal, soil fertilizer, etc. (Katakojwala et al., 2020). Among all the experimental conditions, H-HTL at 250°C showed a cracking effect towards the formation of bio-crude with reduced char formation.

#### 3.3.2 Bio-crude

##### 3.3.2.1 Bio-oil

The major contents of bio-oil constitute combinations of hydrocarbons, alcohols, phenols, acids, esters, ketones, and other chemicals (Li et al., 2022). The bio-oil composition was analyzed by GC-MS and the relative components were calculated according to the peak areas (Table 2). The bio-oil composition predominantly showed aliphatic/aromatic hydrocarbons, carboxylic acids, furan, ketones, and indanone derivatives. N-HTL resulted in lower bio-oil fractions of 7.5% (150°C), 16% (200°C), and 21% (250°C). Although the inert gas (N_2_) did not participate in the reactions, it increased the starting pressure to accelerate the HTL reaction rate by promoting DAB solubility and lowering diffusion resistance, thus decreasing the water viscosity. The total bio-oil fractions in the H-HTL conditions were relatively higher (21% (150°C), 28% (200°C), and 31% (250°C). The higher yields of bio-oil were noticed in H-HTL compared to those in N-HTL indicated that H_2_ acted as a promoter for the hydrogenation reactions. Thus, the hydrodeoxygenation and hydro-denitrogenation reactions can be attributed to the HTL process. H_2_ substitution at a higher temperature (250°C) decreased the levels of unsaturated hydrocarbon and aromatic fractions in the bio-oil by increasing the levels of saturated hydrocarbons. H_2_ in the reactor increases the rate of hydrogenation of unsaturated compounds and the selectivity for aliphatic hydrocarbons by reducing aromatization and polymerization (Hao et al., 2021). Table 3 summarizes the elemental compositions (C, H, N, S, and O), HHV ratios, bio-crude percentages, energy recovery, atom ratios (H/C; O/C; N/C), and elemental enrichment (%) of bio-crude derived at various reaction temperatures and atmospheres. The carbon ratio of bio-oil increased with temperature and atmosphere and oxygen content was significantly reduced, likely due to the deoxygenation reactions under reduced conditions. The HHV of bio-oil increased with temperature and pressure, from 26.5 to 29.4 MJ/kg, which was relatively lower compared to those of fossil-based crude (HHV 42.7 MJ/kg) (Carpio et al., 2021). The energy recovery ranged between 31.58 and 73.01%, with the bio-crude percentages ranging from 25.5 to 53% and the elemental enrichment maximizing (C-65.67%; H-74.8%; N-33.4%; O-35.8%) in H-HTL at 250°C (Table 4). These findings were consistent with those of other studies reporting that increased temperatures under reactive atmospheres lead to deamination reactions during HTL (Maddi et al., 2016; Hao et al., 2021). The elemental ratios of H/C, O/C, and N/C are shown in Table 4. During deoxygenation, the oxygen from the biomass could be eliminated as H_2_O molecules through dehydration or CO_2_/CO via decarboxylation reactions, while nitrogen was eliminated as NH_4_^+^ via deamination (Carpio et al., 2021). At higher temperatures, denitrogenation predominantly occurred with an increased O/C ratio, possibly due to repolymerization, condensation, and cyclization reactions between intermediate compounds that formed at elevated temperatures (Carpio et al., 2021). Overall, deoxygenation tended to occur with increased reaction temperatures, while an H_2_ reaction atmosphere promoted the repolymerization of fragments and resulted in bio-oil fractions. The resulting HTL bio-oil can be converted to aviation fuels (AF) by hydrotreatment/upgradation methods. Upgradation by hydrogenation for bio-oil under H_2_ pressure results in saturated/hydrogenated bio-oils, while hydrocracking yields higher-chain alkanes to desired C_6_-C_15_ hydrocarbons (Basar et al., 2021). These fuels can also be used to replace or blend bio-oil with AF/jet fuel fractions to reduce dependency on THE fossils (Basar et al., 2021).

**TABLE 3:** Conversion effects of HTL process variation with respect to element and relative yields.

Composition	Elemental ratio (%)					HHV (MJ kg-1)	Bio-crude (%)	Energy recovery (%)	H/C atom ratio	O/C atom ratio	N/C atom ratio
		Carbon	Hydrogen	Nitrogen	Sulphur							Oxygen
Initial DAB		48.1	6.3	9.5	0.5	35.6	21.39	-	-	1.56	0.55	0.17
150°C	N_2_	53.5	8.5	7.8	0.69	29.45	26.50	25.5	31.58	1.89	0.41	0.12
	H_2_	53.2	8.6	8.1	0.61	30.1	26.46	47.1	58.26	1.92	0.42	0.13
200°C	N_2_	56.4	8.9	7.9	0.74	26.5	28.29	41	54.22	1.88	0.35	0.12
	H_2_	57.6	8.6	6.8	0.4	27	28.14	52	68.4	1.77	0.35	0.1
250°C	N_2_	58.5	8.6	7.3	0.5	25.1	28.68	44.1	59.13	1.75	0.32	0.1
	H_2_	59.6	8.9	6.1	0.61	24.1	29.47	53	73.01	1.77	0.30	0.08

**TABLE 4:** HTL effects on elemental enrichment (%).

Composition	Element enrichment, %			
Initial DAB		Carbon	Hydrogen	Nitrogen	Oxygen
At 150°C	N_2_	28.36	34.4	20.9	21.0
	H_2_	52.09	64.3	40.1	39.8
At 200°C	N_2_	48.0	57.9	34.1	30.5
	H_2_	62.27	70.9	37.2	39.4
At 250°C	N_2_	53.63	60.2	33.8	31.0
	H_2_	65.67	74.8	33.4	35.8

##### 3.3.2.2 Aqueous fraction (HTL-AF)

The aqueous soluble fraction of bio-crude accounted for 18–26% of the total feedstock. The compositional spectrum was analyzed by HR-MS. Figure 4B presents the detailed components and relative composition of HTL-AF. During HTL, N-HTL resulted in organic soluble fractions in the range of 1088 ppm (150°C), 1555 ppm (200°C), and 2204 ppm (250°C). H-HTL resulted in 2989 ppm (150°C), 4287 ppm (200°C), and 5190 ppm (250°C). Unlike the bio-oil fraction, aqueous-soluble hetero compounds were observed in the HTL-AF fractions due to their higher solubility. The N-hetero atom compounds in the ranges of 123 ppm (150°C), 256 ppm (200°C), and 329 ppm (250°C) were observed in the N-HTL conditions. In contrast, H-HTL resulted in 695 ppm (150°C), 1187 ppm (200°C), and 1408 ppm (250°C). The N-hetero atom compounds might be generated due to deamination and dehydrogenation mechanisms that degrade proteins and amino acid components (Maddi et al., 2016; Hau et al., 2021). The aqueous stream also contained organic oxygenates such as xylose, butyrolactone, angelica lactone, furfurals, cellobiose, levulinic acid, fructose, levoglucosenone, etc. and carboxylic acids like formic acid, acetic acid, glycol acid, etc. as larger fractions (Figure 4B). These were produced by the degradation of carbohydrate and hemicellulose fraction of biomass. Additionally, increased reduction reactions occurred in presence of the H_2_ atmosphere, monosaccharides, and other derivatives, which might result in the formation of xylose, furfurals, levoglucosenone, and butyrolactone. The short-chain carboxylic acids were formed due to the isomerization and hydrolysis reactions of monomers (Reddy et al., 2016; Kopperi et al., 2022). Since HTL-AF contains considerable fractions of organic matter, it can be integrated with energy/nutrient recovery models for resource recovery.

#### 3.3.3 Gaseous fraction

After the HTL reaction, gases from the reactor headspace were collected and analyzed. Gaseous products such as H_2_, CH_4_, CO_2_, and others at lower fractions (possibly ammonia, C_2_H_6_, CO, and C_3_H_8_) were observed. The reactive temperature and atmosphere might have shown a greater influence on the gas profiles (Figure 4C). Under nitrogen atmospheric (N-HTL) conditions, N-HTL at 150°C produced 2.8% H_2_, 1.52% CH_4_, 3.1% CO_2_, and 0.5% others. N-HTL at 200°C produced 4.2% H_2_, 1.5% CH_4_, 3.6% CO_2_, and 1.7% others. N-HTL at 250°C yielded 4.8% H_2_, 2.66% CH_4_, 4.9% CO_2_, and 2.5% others. The variations in HTL temperature implied that water acted as a catalyst that aided in the formation of CO_2_, CH_4_, and H_2_ as the major gases, mainly by promoting decarbonization and water-gas shift reactions (Hao et al., 2021). The other gases from the reaction could be produced from the cracking effect of longer-chain alkanes (Hao et al., 2021). Similarly, H-HTL at 150°C produced 92.2% H_2_, 1.52% CH_4_, 3.1% CO_2_, and 0.5% others. H-HTL at 200^°^C produced 88.6% H_2_, 4.5% CH_4_, 3.5% CO_2_, and 3.2% others. H-HTL at 250°C yielded 86.3% H_2_, 5.2% CH_4_, 4.9% CO_2_, and 3.4% others. In the present study, H_2_ acted as a reducing atmosphere to initiate faster reactions and also reacted with DAB during deoxygenation. The significant reduction of pressure indicated higher H_2_ consumption that aided in the removal of heteroatoms in different forms (H_2_O, NH_3_, and CH_4_) (Barreiro et al., 2013; Antonetti et al., 2016). The H_2_ consumption in H-HTL suggested that the reducing atmosphere facilitated the formation of higher hydrocarbon fractions compared to the N-HTL of bio-oil (Table 3).

#### 3.3.4 Influences of temperature and pressure

The yield and selectivity of bio-crude fractions are predominantly influenced by reaction conditions such as reaction temperature, atmosphere, and solvent medium. The rates of re-polymerization, hydrolysis, fragmentation, and other reactions direct the final product formation (Reddy et al., 2016; Katakojwala et al., 2020). During N-HTL, the N_2_ pressure reached maximums of 116 bar (150°C), 121 bar (200°C), and 126 bar (250°C) at the reaction end (60 min), with slight decreases (1–3 bar) upon cooling (90 min) (Figure 4D). Although N_2_ (inert) gas is involved during the chain reaction, the increase in initial pressure accelerated the HTL rates. The H_2_ (H-HTL) atmosphere resulted in maximum values of 127 bar (150°C), 129 bar (200°C), and 131 bar (250°C) by the end of 60 min, with drops of 4–9 bar upon cooling. H_2_ gas acts as a reducing atmosphere, promoting the reaction rate and product selectivity towards hydrocarbons. The bio-crude yields were higher in H-HTL than in N-HTL, indicating the role of H_2_ as a catalytic promoter of hydrogenation. Pressure is also a critical factor in lowering the reaction time and energy consumption for bio-crude formation. The pressurized inert gas and temperature reduce the viscosity and dielectric constant of water by lowering the diffusion resistance (aids in acid-catalyzed reactions by H^+^ concentration) and increase DAB solubility (Duan, and Savage, 2011). Temperatures not only affect the deoxygenation rate of feedstock to bio-crude yield but also prevent byproduct formation by a cracking mechanism. The C and N recovery during HTL bio-crude yields was significantly influenced by the reaction temperature. High temperatures also increase the kinetic energy and collision frequency, which hasten the cracking process by converting longer-chain alkanes to short-chain alkanes and CH_4_ (Reddy et al., 2016; Basar et al., 2021). As the temperature increases, the bio-crude yields increased in both N-HTL (12.75–44.1%) and H-HTL (47–52%) conditions. Further temperature increases might result in gas formation. Similarly, the trends of char formation showed inverse relationships to bio-crude yields in N-HTL (74.5–54.9%) and H-HTL (52.8- 47%). Water acts as a solvent and has advantages for easy bio-oil separation, promotes lipid hydrolysis, shifts the deoxygenation reaction pathway, and inhibits secondary reactions such as ketonization and esterification (Kopperi et al., 2022). The reaction temperature also affects bio-crude formation by varying the polarity of water. A change in temperature increases the ionic production of water (Kw = [H+] [OH]), which liberates more H+ and OH ions to drive the hydrothermal cleavage of DAB biomolecules to bio-crude (Reddy et al., 2016).

### 3.4 Dark fermentation of HTL-AF

**FIGURE 5:**
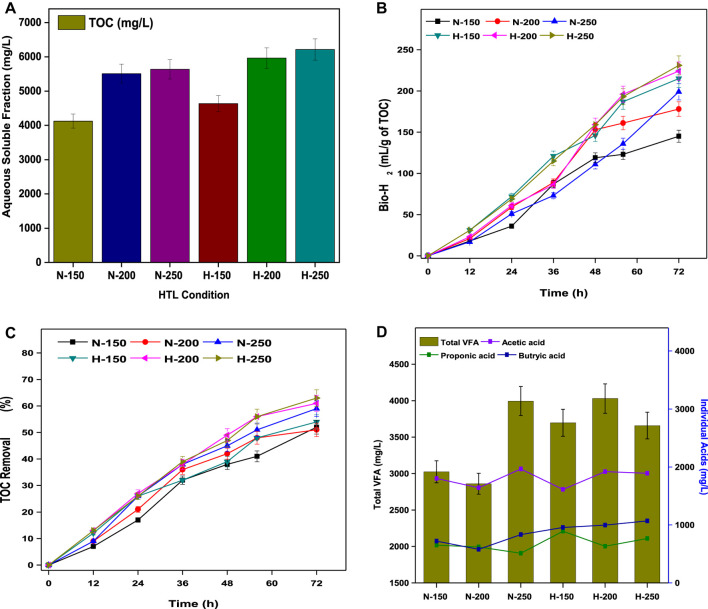
**(A)** Aqueous soluble fraction from the TOC analysis. **(B)** Total bio-H_2_ production. **(C)** Total VFA and individual acid profiles. **(D)** Percentages of TOC removal.

The TOCs of the water-soluble aqueous fractions in both N-HTL and H-HTL were characterized and were tightly related to the HTL conditions. The TOC of N-HTL resulted in 4125 mg/L (N-150), 5510 mg/L (N-200), and 5637 mg/L (N-250), while the TOC of H-HTL resulted in 4637 mg/L (H-150), 5966 mg/L (H-200), and 6216 mg/L (H-250) (Figure 5A). In both conditions, H-HTL resulted in higher carbon flux due to the formation of more polar compounds as they dissolve efficiently in water, leading to the formation of HTL-AF, which showed a significant difference in the composition, as detailed in Section 3.3.2.2. Compared to the lower temperature (150°C), higher total nitrogen and aromatic compounds concentrations were observed at higher temperatures, which were attributed to increased degradation reactions and protein content in the feedstock. The presence of these chemicals could be challenging for the disposal of the water stream due to their toxicity (Si et al., 2019). Thus, nutrient recycling by DF might be a feasible route for detoxification as well as valorization of HTL-AF in the context of biorefinery.

#### 3.4.1 Bio-H_2_ and substrate removal

The enriched biocatalyst dominated the acidogenesis process by proceeding towards acetogenesis and methanogenesis; thus, DF of HTL-AF resulted in bio-H_2_ and VFA production. Figure 5B shows the bio-H_2_ production from HTL-AF of all HTL experimental conditions. The maximum bio-H_2_ yield of TOC conversion (231 ml/g) was observed by the end of 72 h for H-HTL (250°C), with a composition of 55% ± 3 H_2_ and 35% ± 5 CO_2_. Similar performances were observed in all treatment conditions, ranging from 215–231 ml/g of TOC in H-HTL conditions and 178–215 ml/g of TOC in N-HTL conditions. In general, the acidogenesis of 1 mol of glucose produced 4 mol of H_2_ by the acetic acid pathway and 2 mol of H_2_ from the butyrate pathway (Eqs 9, 10).

$$C_{6}H_{12}O_{6}+4H_{2}O\to 2CH_{3}\mathrm{CO}O^{-}+2\mathrm{HC}O_{3}^{-}+4H_{2}+4H^{+}\Delta G^{\text{0}}=-48\mathrm{kJmo}l^{-1}$$ (9)

$$C_{6}H_{12}O_{6}+4H_{2}O\to CH_{3}CH_{2}CH_{2}\mathrm{CO}O^{-}+2\mathrm{HC}O_{3}^{-}+2H_{2}+3H^{+}\Delta G^{\text{0}}=-137\mathrm{kJmo}l^{-1}$$ (10)

Initially (until 12 h), due to hydrolysis, the bio-H_2_ generation was limited, and then increased rapidly until 48 h before stabilizing by end of 72 h. This might be attributed due to a pH drop (4.5) caused by VFA accumulation, which caused *in-situ* acid shock to the biocatalyst, resulting in decreased H_2_ yields. The increased H_2_ yields could be attributed to an acidophilic environment that aided the proton (H+) rich reactions, thus accelerating substrate conversion (Santhosh et al., 2021; Sarkar et al., 2021). The levels of aqueous soluble organic compounds decreased over time through catabolic reactions, resulting in a maximum TOC removal of 63% in H-HTL (250°C) and the overall substrate degradation trend demonstrated in all treatment conditions, ranging from 54–63% in H-HTL and 51–59% in N-HTL (Figure 5C). Although the reaction conditions and substrate concentrations were the same in all experiments, the difference in the HTL-AF product spectrum in each case might be influenced by substrate removal and fermentation products. The acidic pH condition favored acidogenesis and yielded higher VFA production. Analysis of total VFA and its relative composition (acetic acid (AA), butyric acid (BA), and propionic acid (PA)) produced in all experiments showed a maximum total VFA production of 4.03 g/L with a relative composition of 1.92 g/L AA, 0.63 g/L BA, and 0.99 g/L PA in H-HTL (250°C) by the end of 72 h. This and all other experimental VFA profiles are shown in Figure 5D. The total VFA and composition trends were satisfactorily correlated with substrate consumption and bio-H_2_ yields.

### 3.5 Lifecycle assessment of the biorefinery processes

**FIGURE 6:**
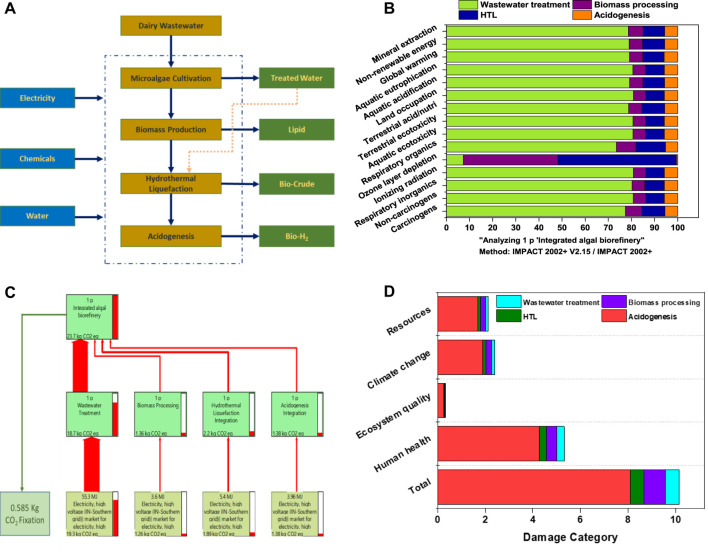
**(A)** System boundary of the integrated algal biorefinery considered in the LCA analysis. **(B)** Lifecycle analysis results of the biorefinery scenarios. **(C)** Sankey diagram representation of the global warming potential. **(D)** Endpoint/damage categories.

The quantified impact categories were derived from the inventory data (Supplementary Table S2). The LCA assessments of the feasibility, system engineering, and environmental impacts of microalgae on biofuels according to various thermochemical pathways showed that the standalone process was energy-intensive and the suggested need for process integration for sustainability (Bennion et al., 2015). The integration process in this study led to the production of lipids, bio-oil, and bio-H_2_. The individual inputs of the processes showed their specific contributions with respect to each mid-point and end-point category. From all inputs, energy in the form of electricity showed its high impact on most of the mid-point categories. Among all unit operations, algal cultivation with the wastewater step showed its higher impact on mid-point categories and its significant share of global warming, land occupation, terrestrial/aquatic ecotoxicity, ionizing radiation, and non-renewable energy. However, during the algal cultivation process, the resulting biomass fixed approximately 0.58 kg of CO_2_ eq by the photosynthetic process, indicating a reduced burden on the environment (Figure 6B). Other steps, such as biomass processing, HTL, and acidogenesis involving various chemicals (solvents), also exhibited lower impacts. The global warming potential (climate change) (IPCC GWP 100a LCIA method) was represented in the Sankey diagram, with the direction of the arrows with specific width proportional to the matter structure and distribution (energy/material) within the integrated system (Figure 6C; Supplementary Table S3). The integration process resulted in the production of 23.7 kg of CO_2_ eq per 100 L, in which >90% (18.74 kg CO_2_ eq) of carbon emissions were released through electricity, which was used during the algal cultivation step, followed by the HTL process (2.1 kg of CO_2_ eq), and the acidogenesis (1.38 kg CO_2_ eq) and de-oiling (1.3 kg CO_2_ eq) steps. The electricity used was predominantly derived from fossil fuels (Coal), resulting in the highest CO_2_ gas emissions. In the analysis of damage categories, algal cultivation operation showed more effects on human health, followed by climate change, resources, and ecosystem quality (Figure 6D). Furthermore, energy from renewable resources may aid in the overall sustainability, with low carbon emissions.

## 4 Conclusion

The results of the present study demonstrated the integrated biorefinery process for nutrient recovery from dairy wastewater using *Coelestrella* sp. and the production of an energy-dense biomass. The removal efficiencies of phosphate and nitrate were 70.39 and 80.1% respectively. The lipid analysis resulted in a higher SFA fraction (50.6%), mostly heptadecanoic acid (C17:0, 11.5%) and a MUFA fraction of 39.6%. The liquefaction of de-oiled biomass resulted in higher bio-oil yields in H-HTL (31%; 250°C) compared to those in N-HTL (21%; 250°C), suggesting the role of H_2_ for hydrogenation. This study also explored the organic oxygenate/acid composition of the aqueous streams of N-HTL and H-HTL in detail. The gas profiles showed 5.2% CH_4_ and 4.9% CO_2_ in H-HTL at 250°C and a decline in pressure at the end of all H-HTL reactions, indicating H_2_ consumption for the formation of higher hydrocarbons. However, further up-gradation to remove oxygen content is recommended to bio-crude to provide an aviation fuel with a higher HHV (42.7 MJ/kg). The HTL-AF valorization suggested that nutrient recycling and bio-H_2_ production by DF could act as a wastewater detoxification process. The LCA of integrated biorefinery demonstrated the specific impact of electricity on the mid-point category. Overall, the results suggested the usefulness of a semi-synthesis approach by integrating biological and HTL conversion processes will accelerate the implementation of circular practices.

## Data Availability

The raw data supporting the conclusion of this article will be made available by the authors, without undue reservation.
