# Supplementary material for: Comparative appraisal of nutrient recovery, bio-crude, and bio-hydrogen production using Coelestrella sp. in a closed-loop biorefinery
Source: Front Bioeng Biotechnol. 2022 Sep 23;10:964070. doi: 10.3389/fbioe.2022.964070 (PMC9537770; doi:10.3389/fbioe.2022.964070)
Supplement: Supplementary file 1 [file Table1.docx]

**Supplementary Information**

**Table 1: GC-MS Product Spectrum of N-HTL and H-HTL of Bio-oil**

| **RT (min)** | **Compound** | **N-150 ^O^C** | **N-200 ^O^C** | **N-250 ^O^C** | **H-150 ^O^C** | **H-200 ^O^C** | **H-250 ^O^C** |
| --- | --- | --- | --- | --- | --- | --- | --- |
| **7.51** | 2-Furancarboxaldehyde | 6.8 | 9.1 | 12.2 | 6.4 | 4.3 | 5.1 |
| **13.69** | Nonane | 2.1 | 2.1 | 5.6 | 6.5 | 1.6 | 0.6 |
| **14.22** | Tetradecane |  | 1.1 | 3 | 3.1 | 0.52 | 0.6 |
| **14.52** | Phenol | 2.2 | 3.1 | 2.3 | - | 0.89 | - |
| **14.61** | Benzoic acid | - | 1.6 | 2 | 1.2 | 3.43 | 1.7 |
| **14.72** | Heptacosane | - | 0.9 | - | 0.6 | 0.92 | 1.2 |
| **15.12** | Dodecanoic acid | - | 0.78 | 1.6 | 2.29 | 1.4 | 0.5 |
| **15.16** | Hexadecane | 3.6 | 5.2 | 5 | 0.91 | 2.04 | 2.3 |
| **15.81** | pentadecane | 1.9 | 1.1 | 1.5 | - | 0.57 | - |
| **16.31** | Heptadecane | 1.2 | 0.3 | 3 | 1.8 | 1.07 | 0.9 |
| **17.33** | Hexadecene | 0.7 | - | 1.5 | 3.01 | - | - |
| **17.29** | Diethyl Phthalate | 2.8 | 0.5 | 0.3 | 0.22 | - | 0.9 |
| **17.51** | Tetradecanoic acid | - | 2.9 | - | 1.7 | 3.32 | - |
| **17.59** | Octadecene | 3.9 | 1.8 | 2.1 | 2.6 | 3.75 | 0.17 |
| **17.81** | Octadecane | - | 0.56 | 0.3 | 7.4 | 1.51 | 0.8 |
| **18.31** | Decane | 0.8 | 2.2 | 4.3 | 1.6 | 8.3 | 1.6 |
| **18.51** | Pentadecanone | 5.1 | 1.1 | - | - | - | 0.6 |
| **18.63** | Cyclononasiloxane | - | 3.5 | 2.6 | 0.2 | 0.66 | 4.51 |
| **18.71** | Nonadecane | 0.51 | - | 1.2 | 3.1 | 0.75 | 1.67 |
| **18.44** | Triacontane | 1.25 | 2.1 | - | - | 3.35 | - |
| **19.22** | Heneicosane | - | 1.1 | 0.5 | 1 | 1.8 | - |
| **19.86** | Propenal | - | 1.3 | 2.9 | 2.5 | 6.1 | 9.1 |
| **20.14** | Isopropyl palmitate | 5.1 | 0.6 | 2.6 | 0.59 | 0.93 | - |
| **20.49** | Hexadecanoic acid | 4.75 | 6.6 | 1.5 | 1.81 | 6.41 | 12.1 |
| **20.62** | Heptadecanoic acid | - | 2.5 | 10.1 | 2.3 | 9.1 | 3.3 |
| **21.13** | 1,2-Benzenedicarboxylic acid | 2.9 | 0.9 | 4.5 | 1.2 |  |  |
| **21.29** | Eicosane | 11.65 | 4.1 | 2.1 | 2.1 | - | 1.9 |
| **21.63** | Octadecanoic acid | 2 | 4.6 | 2.9 | 1.9 | 2.7 | 6.3 |
| **22.97** | Methylene chloride | - | 1 | 23 | - | - | 1.1 |
| **23.23** | Docosane | 1 | 2.3 | 0.6 | 3.6 | 4.9 | 2.2 |
| **24.61** | Phthalic acid | 1.1 | 2.1 | 4 | 1.02 | - | 1.3 |
| **28.81** | Cyclohexanone | 6.8 | - | 1 | 0.6 | - | 2.16 |
