# Supplementary material for: Comparative appraisal of nutrient recovery, bio-crude, and bio-hydrogen production using Coelestrella sp. in a closed-loop biorefinery
Source: Front Bioeng Biotechnol. 2022 Sep 23;10:964070. doi: 10.3389/fbioe.2022.964070 (PMC9537770; doi:10.3389/fbioe.2022.964070)
Supplement: Supplementary file 2 [file Table2.docx]

**Table 2: Inventory data of integrated biorefinery for life cycle assessment analysis (LCA)**

| **Sl No** | **Microalgae cultivation** | | **Algae De- oiling** | | **Hydrothermal Liquefaction** | | **Acidogenesis** | |
| --- | --- | --- | --- | --- | --- | --- | --- | --- |
|  | **Input parameters** | | | | | | | |
| 1 | Dairy wastewater | 100 L | Algae biomass | 320 g | Algae biomass | 240 g | HTL aqueous fraction | 40 g |
| 2 | Electricity | 55.3 MJ | Electricity | 3.6 MJ | Water | 160 g | Water | 4 L |
| 3 | CO_2_ | -585.6 g |  |  | Hydrogen | 7.12 g | Electricity | 3.96 MJ |
|  |  |  |  |  | Electricity | 5.4 MJ |  |  |
| **Output parameters** | | | | | | | | |
| 1 | Biomass | 320 g | Lipid | 83.2 g | Bio-oil | 74.4 g | Bio-H_2_ | 1848 mL |
| 2 | Treated water | 95 L | DAB | 236.8 g | Aqueous fraction | 50.4 g | Volatile Fatty acids | 28.32 g |
| 3 | Oxygen | 425.6 g |  |  |  |  |  |  |
