# Supplementary material for: Comparative appraisal of nutrient recovery, bio-crude, and bio-hydrogen production using Coelestrella sp. in a closed-loop biorefinery
Source: Front Bioeng Biotechnol. 2022 Sep 23;10:964070. doi: 10.3389/fbioe.2022.964070 (PMC9537770; doi:10.3389/fbioe.2022.964070)
Supplement: Supplementary file 3 [file Table3.docx]

**Table 3: LCA results of integrated biorefinery process on different mid-point impact categories using Impact 2002+ method**

| **Impact category** | **Unit** | **Total** | **Integrated algal biorefinery** | | | |
| --- | --- | --- | --- | --- | --- | --- |
|  |  |  | **Wastewater Treatment** | **Biomass Processing** | **Hydrothermal Liquefaction** | **Acidogenesis** |
| Carcinogens | kg C2H3Cl eq | 0.12018021 | 0.092986976 | 0.0084403 | 0.012093715 | 0.006659224 |
| Non-carcinogens | kg C2H3Cl eq | 0.49089575 | 0.39637999 | 0.02652218 | 0.03960699 | 0.028386588 |
| Respiratory inorganics | kg PM2.5 eq | 0.051403207 | 0.041264983 | 0.002805201 | 0.004377848 | 0.002955175 |
| Ionizing radiation | Bq C-14 eq | 89.743874 | 72.523393 | 4.8206018 | 7.2061461 | 5.1937326 |
| Ozone layer depletion | kg CFC-11 eq | 3.32E-06 | 2.42E-07 | 1.36E-06 | 1.70E-06 | 1.74E-08 |
| Respiratory organics | kg C2H4 eq | 0.003039751 | 0.002233579 | 0.000257825 | 0.000388391 | 0.000159957 |
| Aquatic ecotoxicity | kg TEG water | 2009.9497 | 1620.9049 | 109.49017 | 163.47424 | 116.08043 |
| Terrestrial ecotoxicity | kg TEG soil | 455.8315 | 367.6405 | 24.834872 | 37.027702 | 26.328421 |
| Terrestrial acid/nutri | kg SO2 eq | 0.39604429 | 0.31194727 | 0.022796802 | 0.03896024 | 0.022339974 |
| Land occupation | m2org.arable | 0.22411749 | 0.18116755 | 0.012012004 | 0.017963695 | 0.012974239 |
| Aquatic acidification | kg SO2 eq | 0.10757055 | 0.085204815 | 0.006212684 | 0.010051141 | 0.006101907 |
| Aquatic eutrophication | kg PO4 P-lim | 0.005302777 | 0.004274621 | 0.000289974 | 0.000432056 | 0.000306125 |
| Global warming | kg CO2 eq | 23.687564 | 18.745251 | 1.358612 | 2.1990868 | 1.3846143 |
| Non-renewable energy | MJ primary | 322.79296 | 254.98047 | 18.786006 | 30.766157 | 18.26032 |
| Mineral extraction | MJ surplus | 0.070692248 | 0.055582451 | 0.004532995 | 0.006596287 | 0.003980514 |
